# Supplementary material for: Structural rearrangements of the histone octamer translocate DNA
Source: Nat Commun. 2018 Apr 6;9:1330. doi: 10.1038/s41467-018-03677-z (PMC5889399; doi:10.1038/s41467-018-03677-z)
Supplement: Supplementary file 1 — Supplementary Information(PDF 8579 kb) [file 41467_2018_3677_MOESM1_ESM.pdf]

Structural rearrangements of the histone octamer translocate DNA

Bilokapic *et al.*

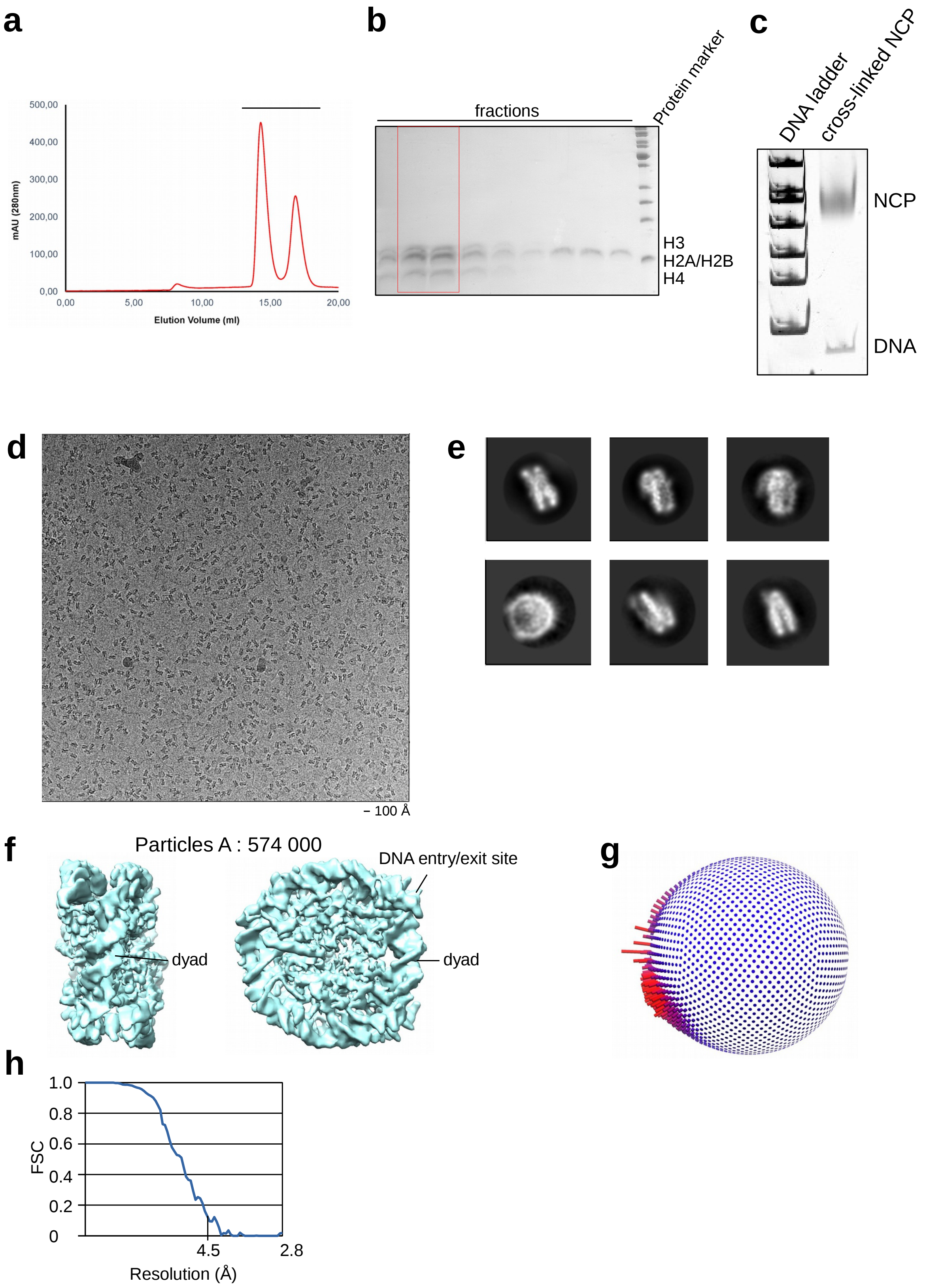

Supplementary Figure 1

Supplementary Figure 1. Cryo-EM reconstruction of the NCP

(a) Chromatogram showing assembly of the histone octamer. Excess of H2A/H2B dimer, used in the octamer assembly, was removed by the size exclusion chromatography (red line).

(b) SDS-PAGE showing assembly of the histone octamer. Selected fractions of the assembled octamer, marked with a black bar on the chromatogram, were analyzed by SDS-PAGE. Fraction marked with red square were taken for the nucleosome reconstitution.

(c) Native gel showing the nucleosome assembly on 149 bp 601 DNA.

(d) Representative cryo-EM raw micrograph collected with Titan Halo electron microscope at 300 keV. NCPs in multiple orientations are clearly visible.

(e) Representative 2D class averages showing the nucleosome core particles in different orientations (side, tilted and top views). Many details are visible in 2D class averages.

(f) Cryo-EM map of the NCP at 4.5 Å (0.143 cutoff in FSC curve).

(g) Angular distribution for cryo-EM map of the NCP.

(h) Fourier shell correlation (FSC) curve showing the resolution of cryo-EM map of the NCP.

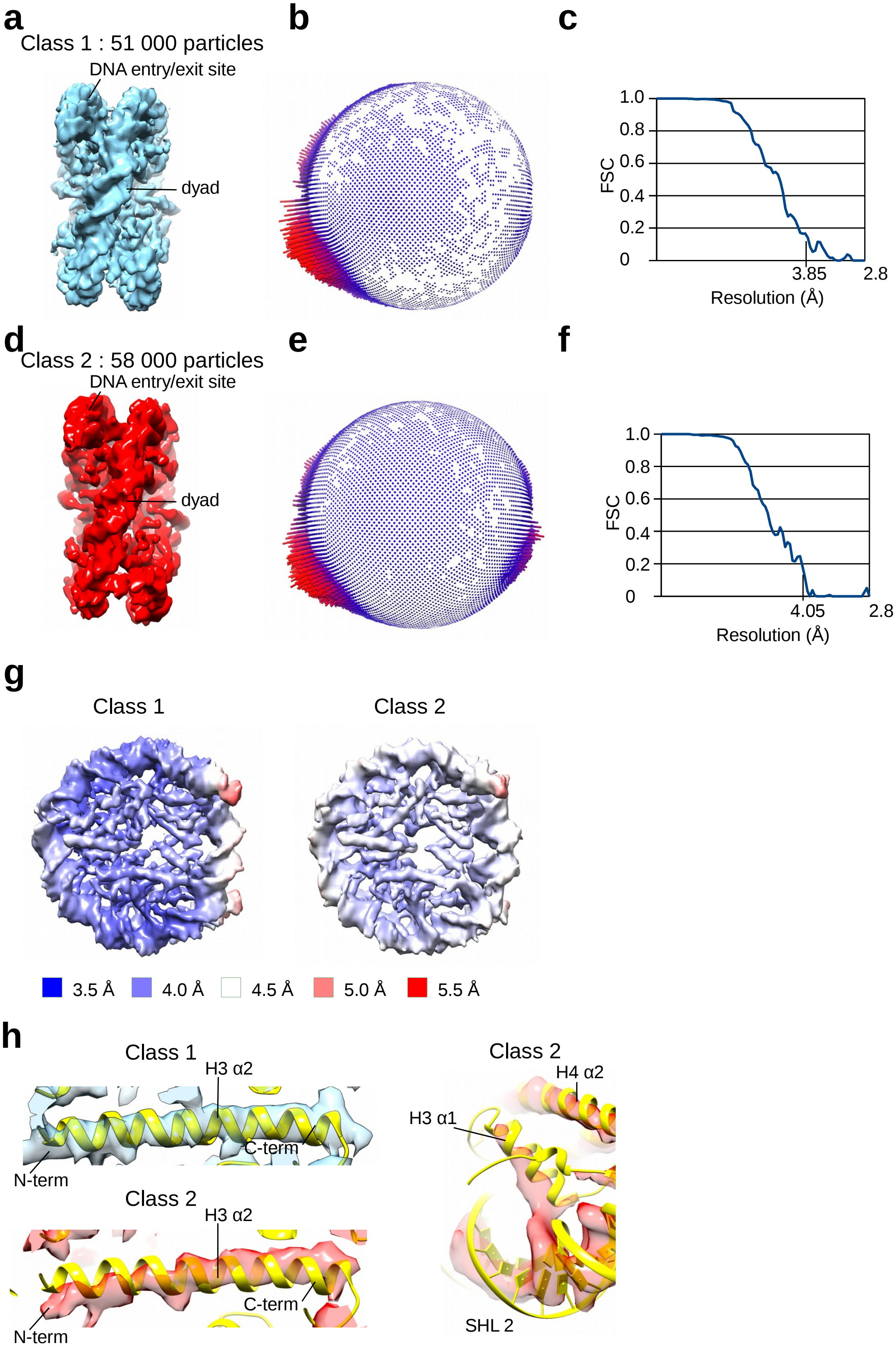

Supplementary Figure 2

Supplementary Figure 2. Classification of the nucleosome core particles

(a) Cryo-EM map of the Class 1 NCP at 3.8 Å (0.143 cutoff in FSC curve).

(b) Angular distribution for the Class 1 cryo-EM map.

(c) Fourier shell correlation (FSC) curve showing the resolution of the Class 1 cryo-EM map.

(d) Cryo-EM map of the Class 2 NCP at 4.0 Å (0.143 cutoff in FSC curve).

(e) Angular distribution for the Class 2 cryo-EM map.

(f) Fourier shell correlation (FSC) curve showing the resolution of the Class 2 cryo-EM map.

(g) Local resolution estimate in Relion.

(h) The H3 α1, H3 α2 and the DNA at SHL 1.5 moved in Class 2. The X-ray model (PDB ID 3LZ1, yellow) does not fit well into the cryo-EM density.

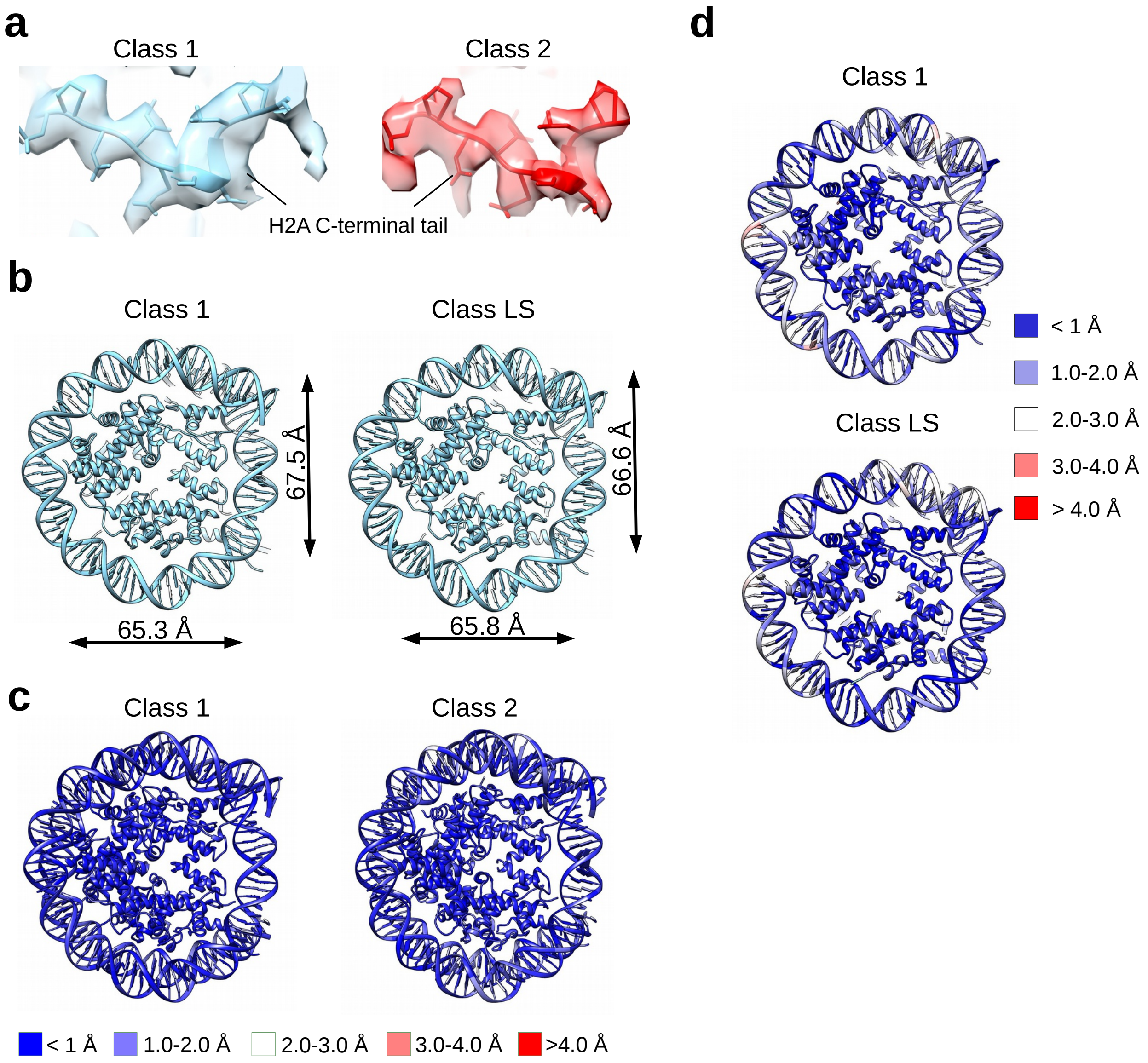

Supplementary Figure 3

Supplementary Figure 3. Fitting of the X-ray model into cryo EM maps

(a) The X-ray structure of the NCP (PDB:3LZ1) was refined into the Class 1 and Class 2 cryo EM maps. Molecular model for each class is fitted to the corresponding cryo EM map showing that side chains are resolved in the histone core. H2A C-terminal tail is shown.

(b) Global changes in the nucleosome structure. Comparison of Class 1 and Class LS models. The nucleosome in Class 1 and Class LS is similar to the X-ray structures.

(c) RMSD (Cα) between the half1 and half2 models for the Class 1 and the Class 2 half models. Deviation between two models from half data sets is less than 0.5 Å for most regions of Class 1 and Class 2 models.

(d) RMSD between the X-ray structure (PDB:3LZ1) and the Class 1 or Class LS models, showing the extent of rearrangements in the NCP. The X-ray structure was superimposed with the Class 1 and Class LS models and RMSD of Cα were calculated and depicted. Class 1 and Class LS are overall similar to the canonical nucleosome structure. Class 1 shows slight distortion.

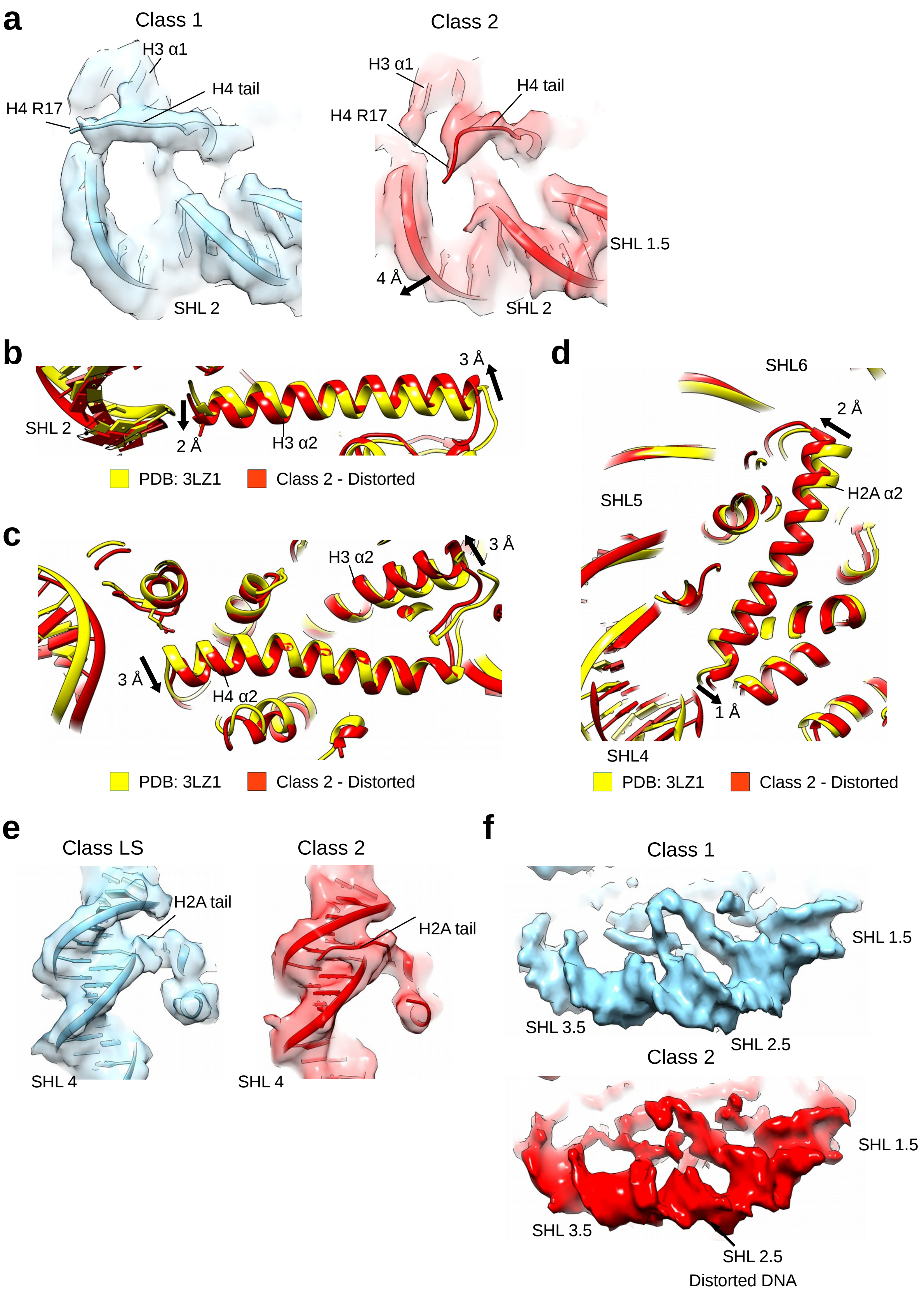

Supplementary Figure 4

Supplementary Figure 4. Structural rearrangements in the histone octamer

(a) In Class LS and Class 1, the H4 N-terminal tail interacts with the DNA phosphate backbone, most likely with positively charged K16 or R17. In Class 2, the DNA slides away more than 4 Å. This detaches the H4 tail from the DNA. In Class 2, the H4 tail is less defined and inserts into the major groove at SHL 2.

(b) Comparison of the X-ray structure (PDB:3LZ1, yellow) and the Class 2 model (red). Arrows depict the direction of the helix movements. The degree of movement between X-ray structure and the Class 2 model is shown, rounded to half an Å.

Conformational rearrangement of the H3 α2. H3 α2 tilts in Class 2 by 2 Å at SHL 2 when compared to the X-ray structure (PDB:3LZ1). This also moves the DNA at SHL 2 for 3 Å outward. At the dyad, the H3 α2 moves 3 Å inward.

(c) Conformational rearrangement of the H4 α2. The H4 α2 bends outward at SHL 2.5 for 3 Å to accommodate for the nucleosome contraction along the symmetry axis.

(d) Conformational rearrangement of H2A α2. The H2A α2 tilts and moves inward 1 Å at SHL 3.5. H2A α2 moves 2 Å outward at SHL 5.5. The DNA at SHL 3.5 is pulled 1 Å in direction of the nucleosome center. At SHL 5 the DNA is pushed 2 Å away from the nucleosome center.

(e) The H2A N-terminal tail is flexible in the Class LS. In Class 2, the H2A tail is inserted into SHL 4.5. In Class 2, histone octamer contracts along the symmetry axis.

(f) Close up view at SHL 2.5 in cryo EM maps. In the Class 1 map, the DNA at SHL 2.5 is defined and resembles a B-DNA helix. In the Class 2 map, the DNA at SHL 2.5 is undefined and does not resemble a B-DNA helix indicating distortion of the DNA. At SHL 1.5 and SHL 3.5, the DNA in Class 2 map resembles a B-DNA helix.

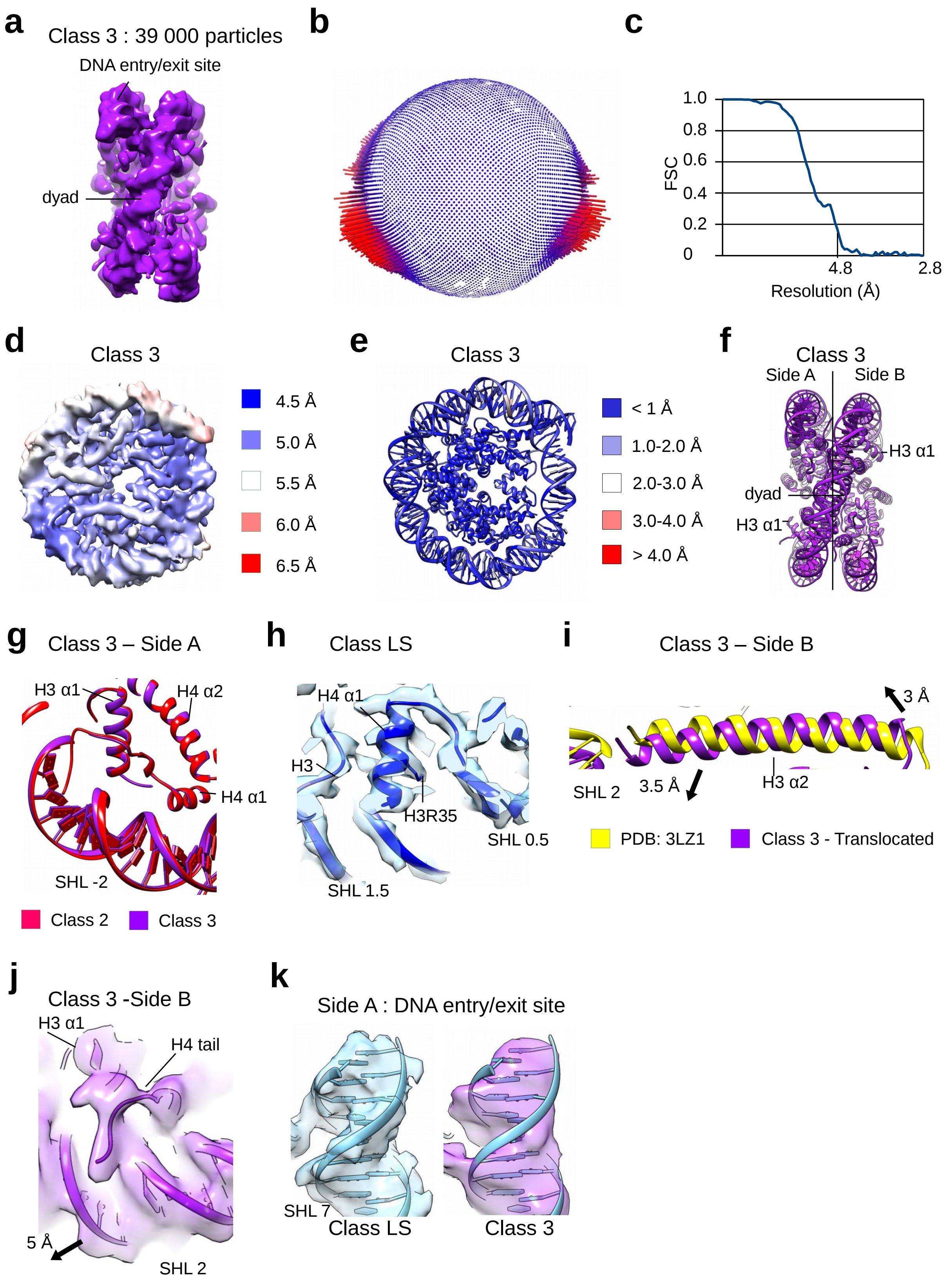

Supplementary Figure 5

Supplementary Figure 5. Conformational changes in the histone octamer translocate DNA

(a) Cryo-EM map of the NCP Class 3 at 4.8 Å (0.143 cutoff in FSC curve). Class 3 contains 39 000 particles. The NCP is shown in purple.

(b) Angular distribution for the Class 3 cryo-EM map.

(c) Fourier shell correlation (FSC) curve showing the resolution of the Class 3 cryo-EM map.

(d) Local resolution estimate in Relion for Class 3 cryo EM map.

(e) RMSD (Cα) between the half1 and half2 models for the Class 3 half models. Deviation between two models from half data sets is less than 1 Å for most regions of Class 3 models.

(f) Side A and B are depicted on the Class 3 model.

(g) Comparison of the nucleosome models for cryo EM maps Class 2 (red) and Class 3, side A (purple).

(h) Fitting of the Class LS (blue) model into cryo EM map. The Class LS map is shown in blue. The H4 α1 interacts with the DNA at SHL 1.5. H4R35 does not make a contact with the DNA at SHL 0.5.

(i) Comparison of the X ray structure (PDB:3LZ1, yellow) and the Class 3 model (purple). Arrows depict the direction of the helix movements. The H3 α2 tilts 3.5 Å at SHL 2.5 and 3 Å in the opposite direction at the dyad.

(j) In Class 3, the DNA slides away more than 5 Å. This detaches the H4 tail from the DNA. Similar to Class 2, in Class 3 H4 tail inserts into the major groove at SHL 2.

(k) The model for Class LS_C1 (Class LS reconstructed with C1 symmetry) was fitted into Class LS_C1 and Class 3 cryo EM maps. DNA entry/exit site of Class 3 and Class LS is shown.

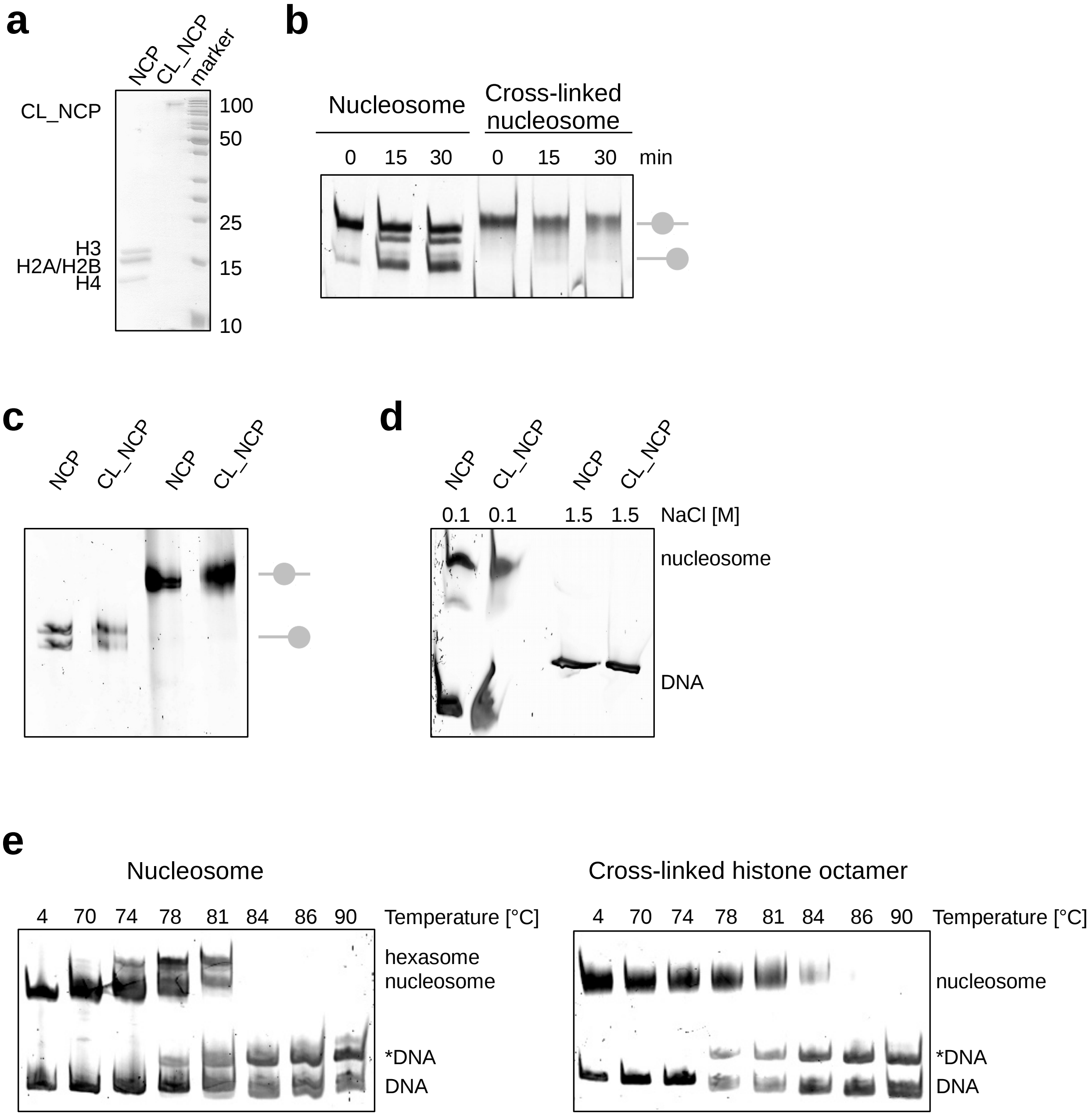

Supplementary Figure 6

Supplementary Figure 6. Conformational rearrangement of the histone octamer is required for the nucleosome sliding

(a) SDS PAGE showing that the histone octamer is cross-linked and migrates at ~100 kDa.

(b) Thermal mobilization of nucleosomes on 227 bp DNA sequence containing strong positioning 601 sequence in the middle. Native nuclesomes are re-positioned at 60 °C. Nucleosomes with cross-linked histone octamer did not move.

(c) Native gel showing migration of native and cross-linked nucleosomes assembled on 227 bp DNA containing 601 sequence either at the end (0-601-80; left) or in the center (40-601-40; right). Cross-linking of the nucleosome does not change the nucleosome migration.

(d) Salt induced disassembly of native and cross-linked nucleosomes assembled on 227 bp 601 DNA sequence. Cross-linked nucleosomes disassemble at elevated salt concentration indicating that histone octamer did not cross-link with the DNA.

(e) Temperature induced disassembly of native and cross-linked nucleosomes assembled on 149 bp 601 DNA sequence. Both, native and cross-linked nucleosomes, disassemble at increased temperature.

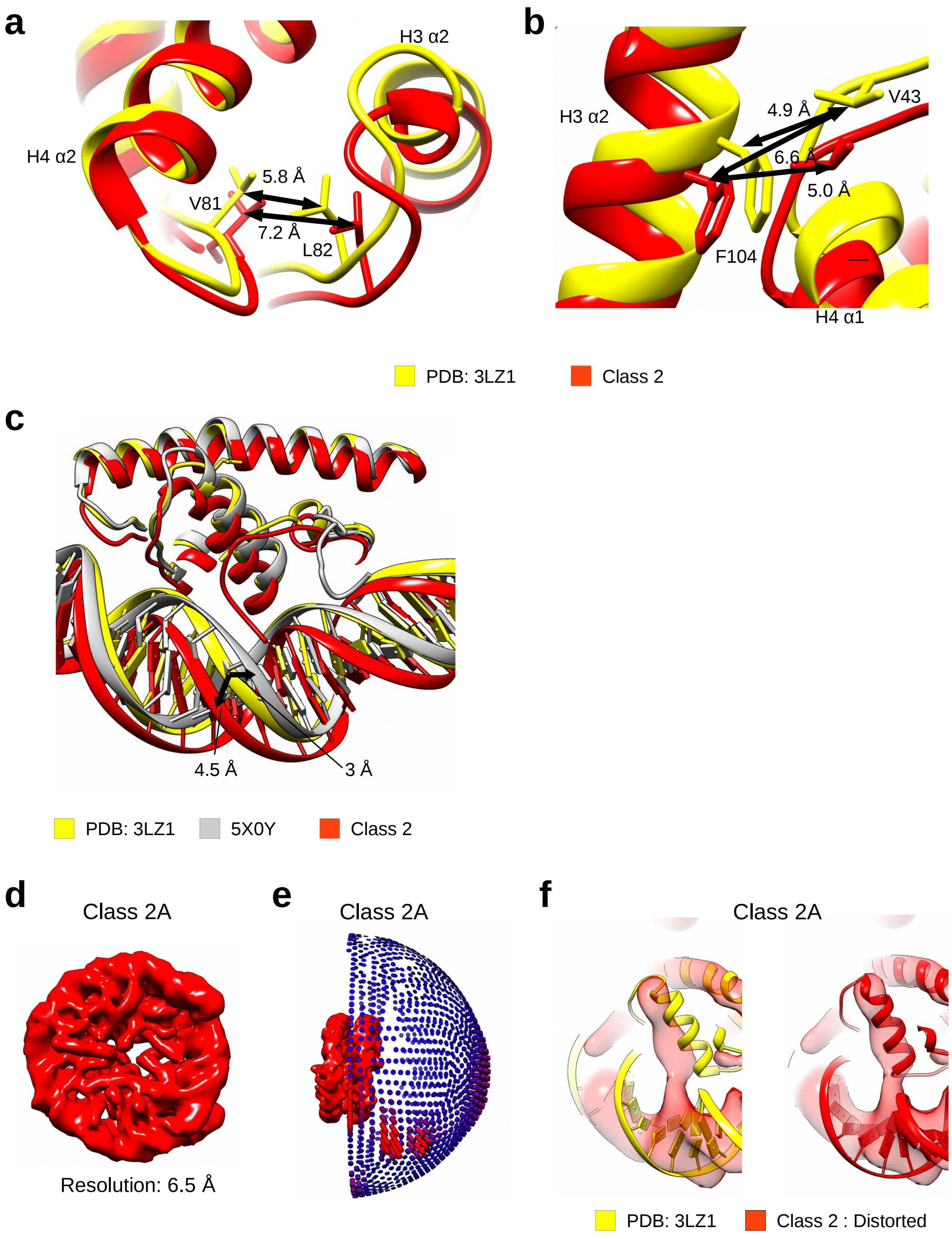

Supplementary Figure 7

Supplementary Figure 7. Comparison with existing data

(a) The distance (Cβ) between H3 L82 and H4 V81 is 5.8 Å in the X-ray structure. In the Class 2 map, the distance is increased to 7.2 Å.

(b) The distance (Cβ) from H3 F104 to H4 V43 is 5.0 Å in the X-ray structure. In the Class 2 map, the distance is increased.

(c) Comparison of the DNA movement between the X-ray, the Class 2 model and the Snf2-Nucleosome complex model (5X0Y). In the Class 2 structure we observe lateral movement of the DNA at SHL 2, while Snf2 pulls the DNA in perpendicular direction.

(d) Cryo-EM map of the NCP Class 2A at 6.5 Å (0.143 cutoff in FSC curve). Class 2A has 50 % of particles in disk orientation.

(e) Angular distribution for the Class 2A cryo-EM map. 50 % particles are in the disk orientation.

(f) The H3 α1 and the DNA at SHL 1.5 (PDB ID 3LZ1, yellow) moved in the Class 2A map and the X-ray model does not fit well. The Class 2 model fits well in the Class 2A map. This excludes that distortion might be caused by the orientational bias.

Figure 5a

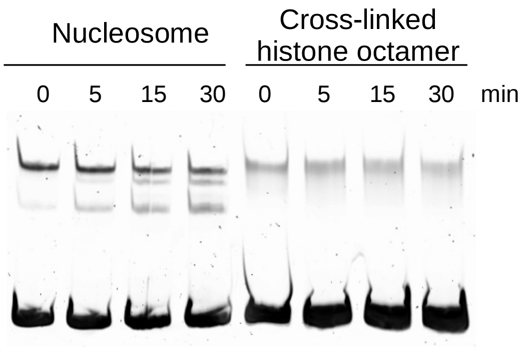

Figure 5d

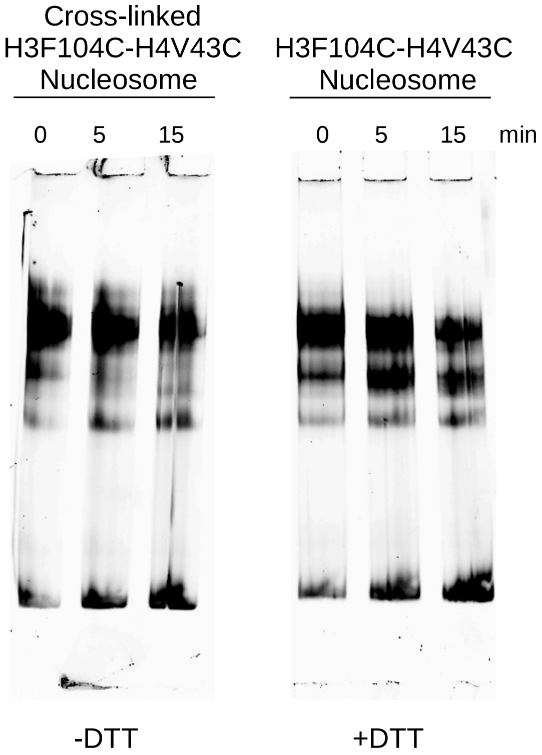

Supplementary Figure 6b

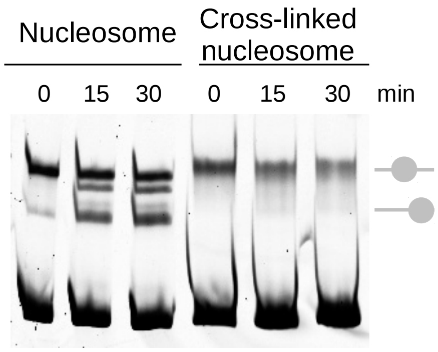

Supplementary Figure 6c

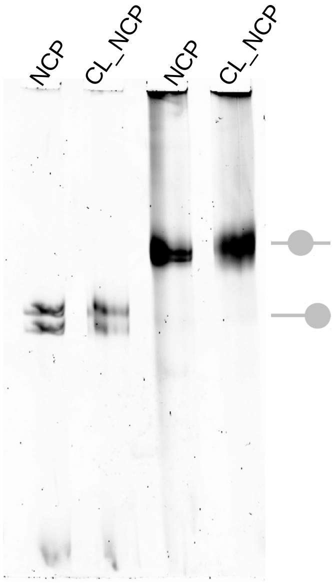

Supplementary Figure 8

Supplementary Figure 8. Unprocessed gels

Supplementary Table 1: Oligonucleotides used in this study

OLIGONUCLEOTIDES	SEQUENCE
149 bp 601 DNA	
423F	GCACAGGATGTATATATCTG
423R	CTGGAGAATCCCGGT
227 bp 601 DNA (+- 40 bp on each side)	
797F	TATCCGACTGGCACCG
797R	GAGTTCATCCCTTATGTGAT
227 bp 601 DNA (+80 bp on one side)	
423F	GCACAGGATGTATATATCTG
1228R	GGATCCTAATGACCAAGG
